# Family Health

**Published:** 1875-02

**Authors:** 


### FAMILY HEALTH.

A considerable percentage of the income of an average family in the United States is expended in patent medicines, doctors' bills, and in the effort to get well, including various appliances, excursions, horse and carriage hire, and visiting distant points of the country, more or less celebrated for one form or other of disease and suffering. "All that a man hath will he give for his life," and, with equal truth, it may be said, "All that a man hath will he give for his health." One of New York's most honorable and retired merchants said, with great earnestness, "I would give half I have if I could only sleep as well as I could when I was a young man," and he was worth a million of money.

There is another item of loss in connection with the pursuit of health, not only the loss of the sufferer's time, but that of other members of the family, who must devote a part of their own, in services to the invalid. Besides this, many have to break up their business, in order that they may leave home for more salubrious climes; others have to change their occupations, to embark in something more healthful; and going into a new business means failure, nine times out of ten. Who does not know that many a life has been blighted by having the health injured while young? In the vast majority of cases the seeds of consumption and dyspepsia are implanted in the system during the "teens" of life.

One half of all the children born, even in civilized society, die before the age of ten years. The average duration of life is forty-two years; it ought to be over three score. Two persons out of three who die in New York City, perish twenty years before their time by avoidable diseases; maladies which would not have invaded the system, if the laws of life had been known and observed; if the persons had only understood how to take care of their health. To impart such knowledge to families, is the object of this publication. I heard a young man from Maine, who was a permanent invalid from gross negligence of himself, curse the memory of his father for never having given him any information on the subject of taking care of himself. "If mother had only told me something about it," has been the despairing exclamation of many a daughter suffering hopelessly.

As the country grows older, and the population more dense, a greater number of persons must live under the same roof; and, in proportion, the necessity of increased care of the health becomes more and more imperative, and greater efforts must be made to impart general information on the subject; the people must know more about

Locating Farms and Houses,
The Warming of Dwellings,
The Ventilation of Apartments,
The Necessity of Cleanliness—
In Habitation,
In Surroundings,
In Clothing,
In Sleeping Apartments,
Procuring Healthful Food,
Preparation of Food,
Methods of Exercise,
Adaptations of Sleep,
Care of Children,
Their Healthful Education,
Selecting Occupations,
How to Maintain Health,
How to Avoid Ordinary Disease, etc.

It is our purpose to give instruction and reliable information as to all these points, from time to time, and, as far as possible, to meet a want in every household—what to do in cases of sudden and alarming sickness or accident, in the country, away from physicians, and in the town at midnight, and in inclement weather.

It is not intended to instruct families in the practice of medicine, in the use of drugs; that is the peculiar and the fitting province of only the educated physician. It is not proposed now, nor have we in the monthly issues of the past twenty years, advised taking a single dose of medicine, except in the one single case of cholera, which is often made fatal by an hour's delay. We shall confine ourselves to external applications, and to the internal use of those domestic remedies which are almost certainly to be found in every kitchen. Some of these we will name as examples, premising that in "Health at Home," or Hall's Family Doctor, 846 pp., 8vo., price $5.00, many pages are devoted to the same kind of information.

Has scalding water fallen on a child? It screams dreadfully, both from fright and pain; the whole household is thrown into alarm and commotion; no one has presence of mind enough to do anything; the doctor is an hour or a mile away; the book says, plunge the limb in cold water, relief is instantaneous, and quiet reigns; then, go to the flour barrel, throw the flour on the scalded spot, until no more will stick on; a close cake is formed, all air is excluded; wrap a cloth around, put the child to bed and to sleep; in two or three days the flour falls off and a new skin has grown without any further attention.

Has an overdose of laudanum, or other narcotic poison been taken? Run into the kitchen, take a teaspoonful each of salt and ground mustard, put them into half a glass of water, stir rapidly and down with it; as soon as it touches the stomach, the entire contents are heaved up. Then, lest there might be a little left, swallow the white of one egg or drink a cup of strong coffee, and all is safe.

In no less than ten different forms of disease is common hog's lard a safe, mild, and at the same time, an efficient remedy.

Surely it must be worth a few cents a month, to have information of such a character imparted to a family, each member of which, from one to a dozen, is instructed for life.

Terms, $1.00 a year; five copies for six dollars; fifteen copies for ten dollars. All orders out of the city payable in Post Office Check to W. W. Hall, 234 Broadway, New York. Hall's Journal of Health, $2.00; both $2.25.

---

### DR. W. W. HALL'S

### HEALTH LIBRARY.

BY THE EDITOR OF HALL'S JOURNAL OF HEALTH.

| Title. | Price each. | No. sold. |
|---|---|---|
| Health Tracts, 350 in number | $2 00 | 1,500 |
| Health and Disease, or Constipation | 1 50 | 8,000 |
| Sleep, or Hygiene of the Night | 1 50 | 10,000 |
| Coughs, Colds, and Consumption | 1 50 | 10,000 |
| Bronchitis and Kindred Diseases | 1 50 | 12,000 |
| Health by Good Living | 1 50 | 25,000 |
| Fun and Physic, or 1,200 Health Maxims | 1 50 | 19,000 |
| Guide to Health, Happiness, and Longevity | 5 00 | 20,000 |
| "Health at Home;" or, Hall's Family Doctor | 5 00 | 12,000 |
| Soldier Health | 5 00 | 41,000 |

**Books sent by mail, 25 cents additional.**

Over 175,000 of Dr. Hall's books on health have been sold, showing an encouraging progress in the tastes of the people for health publications; and an increasing appreciation of the importance of taking care of the health, as it is manifold easier to keep well than to get well.

